# Selection and Validation of Reference Genes for Gene Expression Analysis in *Vigna angularis* Using Quantitative Real-Time RT-PCR

**DOI:** 10.1371/journal.pone.0168479

**Published:** 2016-12-16

**Authors:** Chao Chi, Yongqiang Shen, Lihua Yin, Xiwang Ke, Dong Han, Yuhu Zuo

**Affiliations:** Heilongjiang Bayi Agricultural University, Daqing, Heilongjiang, People’s Republic of China; INIA, SPAIN

## Abstract

Adzuki bean (*Vigna angularis*) is one of the most important legume crops in Asian countries like China, Japan and Korea due to its nutritious protein and starch contents. In spite of its economic importance, gene expression analysis system for gene function verification of adzuki bean is still absent. Therefore, reference genes for gene expression analysis based on the quantitative real time PCR (qRT-PCR) were screened in current study. A total of nine general housekeeping genes, including *ACT*, *Fbox*, *ZMPP*, *GAPDH*, *EF*, *PP2A*, *UBC*, *UBN* and *PTB* were evaluated for their expression stability by qRT-PCR in four adzuki bean cultivars, three different tissues, four abiotic stress and one biotic stress. The best group of candidates as reference genes were as follows: *PTB* and *ACT* for different cultivars; *EF* and *UBN* for different tissues; *ACT* and *ZMPP* for biotic stress and waterlogging stress; *Fbox* and *UBC* for salinity-alkalinity stress; *Fbox* and *PTB* for drought stress. Our results will provide a more accurate and reliable normalization of qRT-PCR data in adzuki bean.

## Introduction

Adzuki bean (*Vigna angularis*) is one of the important legume crops of the Ceratotropis subgenus in Asian, under the papilionoid subfamily of the Fabaceae. Due to its sweet taste with high protein and starch content, adzuki bean is widely cultivated and used as traditional food material in East Asia and other areas [1]. To better understand the gene function of adzuki bean, gene expression analysis system of the crop is of significance. Quantitative real-time reverse transcription PCR (qRT-PCR) is an important technique in gene expression analysis, which relies on reference genes for data normalization. However, systematic evaluation of the reference genes used for gene expression analysis of adzuki bean is still lacking to date. In previous studies, 18S rRNA was used as reference gene to normalize the *VaXTHS4*, *VaXTH1* and *VaXTH2 *expression level of adzuki bean treated with hypergravity [2]. All of these studies on adzuki bean gene expression surveyed have used only one single reference gene and no preliminary validations were performed. However, evidence showed that transcriptional levels of commonly used reference genes (e.g. *GAPDH*, *ACT*) may also vary considerably in response to changes in different experimental conditions and/or tissue types [3, 4]. Therefore, a suitable reference gene(s) that is (are) stable under specific experimental conditions needs to be screened, in order to make the results for adzuki bean gene expression accurate.

Quantitative real-time reverse transcription PCR represents a particularly suitable technology catering for this purpose, attributed to its sensitivity, specificity, dynamic range and high throughput capacity [5–8]. Commonly used reference genes include ribosomal RNA 18S rRNA and a number of housekeeping genes, such as those encoding actin (*ACT*), tubulin (*TUB*), polyubiquitin (*UBQ*) and elongation factor 1-α (*EF1*α) [9, 10]. The importance of choosing stable reference genes prompted the development of a set of software packages, such as geNorm, NormFinder and BestKeeper [11]. Different statistical algorithms in the software packages can result in inconsistent ranking of the optimal reference genes, thus the selection of reference genes requires the consideration of all statistical algorithms [12]. Validations of the stability of reference genes have been performed by the three different software packages in many plants, such as *Arabidopsis thaliana* [13], rice [14, 15], *Brachypodium sp*. [16], wheat [17], soybean [18], tomato [19], sugarcane [20] and grape [21]. Therefore all the three software packages were chosen for data analysis in the current study.

The whole genome sequence of recently reported adzuki bean [22] has facilitated genome-wide mining for reference genes. Nine typically used housekeeping genes were considered as candidate reference genes including actin-like protein (*ACT*), zinc-metallopeptidase (*ZMPP*), elongation factor 1-alpha (*EF1-α*), FBOX domain-containing protein (*Fbox*), polyubiquitin-C (*UBC*), ubiquitously expressed nuclear (*UBN*), branched-chain phosphotransacylase *(PTB)*, serine/threonine protein phosphatase 2A (*PP2A*) and glyceraldehyde 3 phosphate-dehydrogenase (*GADPH*). To validate and develop a qRT-PCR method for adzuki bean gene expression analysis, different experimental conditions including four adzuki bean cultivars, three different tissues, four abiotic stress and a biotic stress were applied.

## Materials and Methods

### Varieties and stress treatments

The following varieties of adzuki bean were included in our experiments: Baoqinghong (BQH), Nonganhong (NAH), ZXC136 and ZXC143. Seeds were provided by the National Coarse Cereals Engineering Research Center (NCCERC, China). Plants were grown in a growth chamber under short daytime conditions (8 h light/16 h dark) at 24°C–27°C when fully expanded euphylla (about 10 d after sowing) were collected for gene expression test.

Only BQH were used to perform abiotic stress, biotic stress and different tissue treatment tests. Different tissues including root, stem and fully expanded euphylla (about 10 d after sowing) were then collected for gene expression assays.

Drought stress was induced by stopping watering while the euphylla were fully expanded. Subsequently, euphylla were sampled at 0, 6 and 12 d after drought treatment. For salinity-alkalinity stress, seedlings were treated with 100 mM complex neutral and alkali salts [NaCl:Na_2_SO_4_:NaHCO_3_:Na_2_CO_3_ = 3:3:5:1], and euphylla sampled after treatments at 0, 6 and 12 d. For waterlogging stress, the pots were placed into large black tanks and the seedlings were submerged under the water which was 2 cm above the soil surface. Samples were collected after treatments at 0, 6 and 12 d. For biotic stress, the fully expanded euphylla were inoculated with uredospore of *Uromyces vignae* in a concentration of 10^5^ uredospore per milliliters as described previously [23]. The inoculated leaves were harvested at 0, 6 and 12 d post inoculation (dpi). Each sample with three replicates were collected and quickly frozen in liquid nitrogen and stored at -80°C for RNA extraction.

### RNA extraction and cDNA synthesis

Total RNA was extracted following the manufacturer instructions using Trizol^TM^ Reagent (Invitrogen, Carlsbad, CA) and then treated with RNase-free DNaseI at 37°C for 30 min. Purification with Trizol^TM^ Reagent was repeated to remove DNaseI. Only the RNA with A260/A280 ratio of 1.8–2.1 and A260/A230 ratio > 2.0 were used for subsequent analyses. The integrity of RNA samples was verified by 1.5% agarose gel electrophoresis with ethidium bromide staining. Following the manufacturer’s instructions, strand cDNA synthesis was performed using the RevertAid^TM^ First Strand cDNA Synthesis Kit (Fermentas, Shenzhen, China) and oligo-dT primers in a final volume of 20 μL. All cDNA samples were stored at -20°C and diluted 10-fold prior to being analyzed in qRT-PCR.

### PCR primer design and qRT-PCR analysis

Primers of the nine candidate reference genes for qRT-PCR were designed using Primer Premier V5.0 (Premier Biosoft International, Palo Alto, CA) and the primer sequences are listed (Table 1). Specificity of the primers was tested by performing PCR using cDNA as template. PCR products were analyzed on 1.5% agarose gels. For each primer pair, PCR reaction efficiency estimates were derived from a standard curve generated from a serial of dilution pooled cDNA. Based on average cycle threshold (Ct) values of each five-fold dilution, a standard curve was generated using linear regression. PCR efficiency (E) was achieved by the equation: Efficiency % = (10^(-1/slope)^ - 1) ×100%. PCR conditions were as follows: 95°C for 1 min, 40 cycles of 95°C for 15 s, 55°C for 30 s, and 72°C for 30 s. Finally, dissociation curves were generated by increasing the temperature from 65 to 95°C, stepwise by 0.3°C every 10 s.

**Table 1:** Primer sequences for the selected candidate reference genes.

Gene symbol	Primer sequences [5’-3’] (Forward/ Reverse)	Amplicon length (bp)
ACT	CTAAGGCTAATCGTGAGAA/CGTAAATAGGAACCGTGT	165
Fbox	ACCCTTTCCCTTCTTTCA/TTATCCTATCCCAGCACC	105
ZMPP	CAGCAGGCTATGAACTAC/CAACTTGAAGAGCAGGAA	91
GAPDH	GTCGGAGGCAACATCACC/GTCCAAATGCGGGAACAG	121
EF	CAAGATGGATGCCACTAC/AGGTCCCTTGTACCAGTC	184
PP2A	TGATCTGTTATCCCAATCT/TGACTTGCCAATAAGGGT	108
UBC	AACAATTATGGGTCCTCC/TGAAATACCTTCGTCCTG	120
UBN	TGAGCCAACTGATACGAT/CAAGCACCAAATGAAGTA	164
PTB	AGATGGGAAGAGGAAAGA/AGAAGACAGTATGGAGGACA	98

### Statistical analysis

Ct values of all samples were transformed to relative quantities using the method of 2^-ΔΔCt^ [24], and then exported to geNorm v3.5 [25], NormFinder [26] and BestKeeper [27] for gene expression stability analysis. The geNorm statistical algorithm calculates gene stability (*M* value) with the average of pairwise variations, relying on the principle that the expression ratio of two ideal reference genes should be identical in all samples. By ranking gene stability via stepwise exclusion of the least stable gene, geNorm identified the genes with the lowest *M* values indicating the highest stability and the number of best reference genes. Additionally, Vandesompele *et al*. [25] recommended that genes with lowest value of V_n/n+1_ (V_n/n+1_ < 0.15) are the most stable genes. NormFinder, another algorithm for identifying the optimal normalization gene(s) among a set of candidates, ranks the stability of candidate reference gene expression for all samples with no sub-group determination. According to the analysis, the lowest stability value would be ranked the highest. Finally, the stability rankings of the reference genes from the three different algorithms were integrated, generating an overall ranking according to the geometric mean [28, 29].

### Normalization of the target genes

For normalization experiments, catalase (*CAT*), chitinase (*GLU*) andβ-1,3-glucanase (*CHI*) genes expression responsive to *U*. *vignae* infection of BQH was used. Sample was harvested at 0, 12, 24, 48 and 120 h after inoculation, respectively. RNA extraction and cDNA synthesis were performed as described above. Expression was normalized using four reference gene combinations: (1) the most stably expressed reference gene, (2) the two most stably expressed reference genes combination, (3) three most stably expressed reference genes combination and (4) the least stably expressed gene.

## Results

### Amplification specificity and efficiency

Total RNA extracted from all samples met the requirement for qRT-PCR analysis (S1 Table). Melting curve analysis and agarose gel electrophoresis showed that all primer pairs amplified a single fragment with expected size. No amplification was detectable in the absence of template. The amplification efficiencies for the nine candidate reference genes ranged from 90.23% (*ZMPP*) to 105.84% (*Fbox*), and regression coefficients from 0.9911 (*Fbox* and *UBC*) to 0.9995 (*UBN*) (Table 2).

**Table 2:** Reference gene parameters derived from RT-qPCR analysis.

Gene symbol	TM (°C)	Amplification efficiency (%)	Regression coefficient (R^2^)
ACT	53	94.45	0.9913
Fbox	53	105.84	0.9911
ZMPP	53	90.23	0.9991
GAPDH	57	104.34	0.9916
EF	55	97.24	0.9976
PP2A	52	91.65	0.9986
UBC	52	97.62	0.9911
UBN	51	93.24	0.9995
PTB	54	95.11	0.9987

### Candidate reference genes expression stability analyses

**Fig 1:**
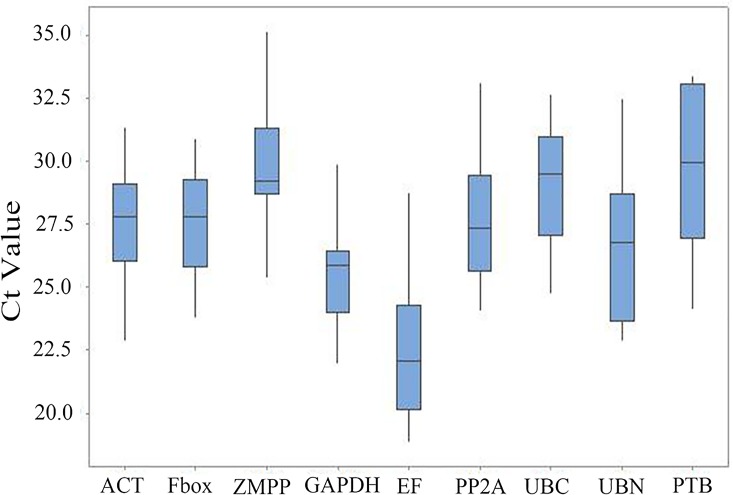
RT-qPCR Ct values for reference genes. Expression data displayed as Ct values for each reference gene in all samples. Depicted is the median. Boxes indicate the 25 and 75 percentiles. Error-bars represent maximum and minimum values.

To show transcriptional differences among various candidate genes, the average Ct value was calculated across all samples. The mean Ct values for nine genes ranged from18.86 to 35.15 (Fig 1). Ct values were transformed to relative quantities using the ΔCt method. NormFinder, geNorm and BestKeeper were applied to calculate the expression stability of the set of candidate reference genes (S2, S3 and S4 Tables).

**Fig 2:**
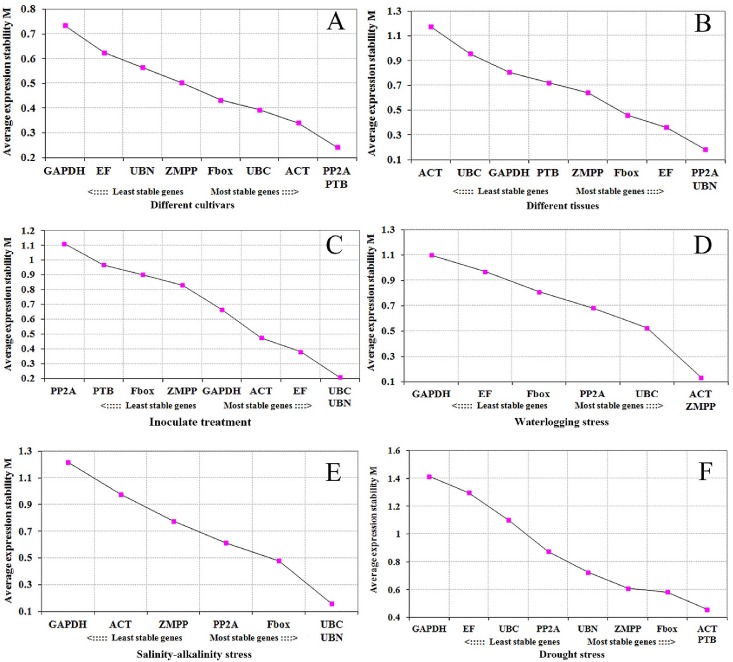
Average expression stability values of reference genes by geNorm analysis. Gene expression values of nine genes across six different treatments were obtained and analyzed using the geNorm software package. Genes were ranked by stepwise exclusion of the least stable gene. Genes with a high *M* value are less stably expressed compared with genes with a low *M* value. (A) Different cultivars, (B) different tissues, (C) inoculation treatment, (D) waterlogging stress, (E) salinity-alkalinity stress, (F) drought stress.

**Fig 3:**
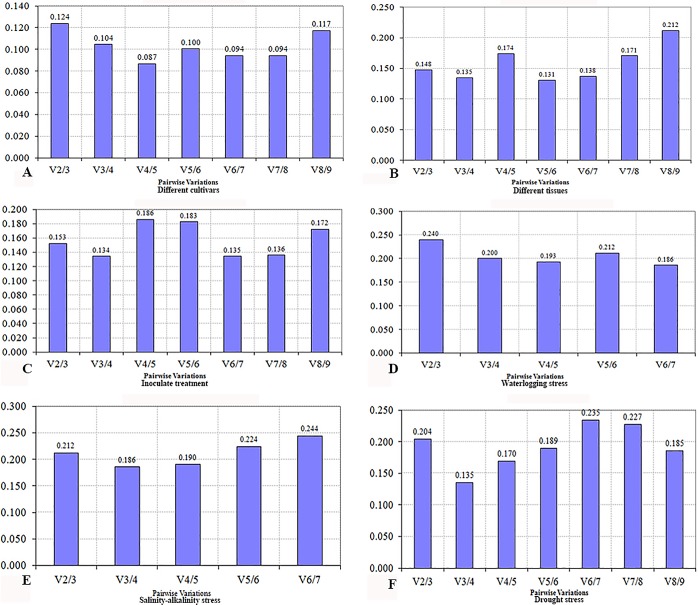
Determination of the optimal number of reference genes by geNorm analysis. Determination of the optimal number of reference genes for accurate normalization of gene expression. Average pairwise variations V_n_/V_n+1_ are calculated between the normalization factors NF_n_ and NF_n+1_ to indicate whether inclusion of extra reference gene adds to the stability of the normalization factor. Each bar represents the change in normalization accuracy when stepwise adding more internal reference genes according to the ranking. (A) Different cultivars, (B) different tissues, (C) inoculation treatment, (D) waterlogging stress, (E) salinity-alkalinity stress, (F) drought stress.

Average expression stability (*M* value) of all genes was calculated by geNorm (S2 Table). Most of the genes used in current study had an *M* value below the geNorm threshold of 1.5 (Fig 2). When challenged with various stresses, different cultivars responded with varying stabilities. Among the various cultivars, *PP2A* and *ACT* remained most stable, and *GAPDH* was least stable (Fig 2A). *PP2A* and *UBN* remained the most stable, and *ACT* the least stable among the various tissues (Fig 2B). In response to rust infection, *UBC* and *UBN* were the most stable, and *PP2A* had the least stability (Fig 2C). When challenged with drought stresses, *ACT* and *PTB* showed the highest stability, and *GAPDH* the lowest (Fig 2D). Responding to salinity-alkalinity stress, *UBC* and *UBN* were the most stable, and *GAPDH* the least (Fig 2E); while in response to waterlogging stress, *ACT* and *ZMPP* were the most stable, and *GAPDH* the least (Fig 2F). To determine the optimal number of reference genes, geNorm was used to calculate the pairwise variation V_n_/V_n+1_ of two sequential normalization factors NF_n_ and NF_n+1_. In various cultivars, two genes were sufficient for normalization, since the V_2/3_ value was < 0.15 (Fig 3). Differences in the expression stability of the candidate reference genes were less marked in the various cultivars treatment series, than in the other series (Fig 3). The V_2/3_ value for the various tissues was 0.148, so that *PP2A* and *UBN* would be sufficient for normalization purposes. With the inoculation treatment, the combination of *UBC*, *UBN* and *EF*, as internal reference genes, produced a V_3/4_ value of 0.134. With the drought stress, the combination of *ACT*, *PTB* and *Fbox* produced a V_3/4_ value of 0.135. However, as for waterlogging and salinity-alkalinity stress, no V_3/4_ values below the threshold were found; and the closest values were 0.200 and 0.186, respectively (Fig 3).

The stability ranking generated by NormFinder was slightly different from that determined by geNorm (Table 3). *Fbox*, *PTB*, *ACT* and *PP2A* were still ranked the highest for various cultivars, and *EF* and *UBN* the highest for different tissue samples, and *ACT* and *ZMPP* the highest for waterlogging stress. *PTB*, *PP2A* and *EF* was the most stable housekeeping genes for drought stress. However, among inoculation treatments, *ACT* and *ZMPP* emerged as the most stably expressed (ranked fourth and sixth by geNorm) and *Fbox* was the most stable for salinity-alkalinity stress.

**Table 3:** Expression stability ranking of the 9 candidate genes.

Method	Ranking (better-good-average)								
	1	2	3	4	5	6	7	8	9
Ranking order under different cultivars									
geNorm	PTB/PP2A		ACT	UBC	Fbox	ZMPP	UBN	EF	GAPDH
NormFinder	Fbox	PTB	ACT	PP2A	UBC	EF	UBN	ZMPP	GAPDH
BestKeeper	ACT	PTB	UBC	UBN	ZMPP	Fbox	PP2A	EF	GAPDH
Comprehensive	PTB	ACT	UBC	Fbox	PP2A	UBN	ZMPP	EF	GAPDH
Ranking order under different tissues									
geNorm	UBN/PP2A		EF	Fbox	ZMPP	PTB	GAPDH	UBC	ACT
NormFinder	EF	UBN	Fbox	PP2A	ZMPP	UBC	ACT	GAPDH	PTB
BestKeeper	Fbox	EF	UBN	ZMPP	PP2A	UBC	GAPDH	PTB	ACT
Comprehensive	UBN	EF	Fbox	PP2A	ZMPP	UBC	GAPDH	PTB	ACT
Ranking order of rust infection									
geNorm	UBC/UBN		EF	ACT	GAPDH	ZMPP	Fbox	PTB	PP2A
NormFinder	ACT	ZMPP	Fbox	PTB	UBC	GAPDH	UBN	EF	PP2A
BestKeeper	ACT	ZMPP	UBC	UBN	EF	PTB	Fbox	GAPDH	PP2A
Comprehensive	ACT	ZMPP	UBC	UBN	EF	Fbox	PTB	GAPDH	PP2A
Ranking order under waterlogging stress									
geNorm	ACT/ZMPP		UBC	PP2A	Fbox	EF	GAPDH	PTB	UBN
NormFinder	ACT	ZMPP	PP2A	UBC	GAPDH	Fbox	EF	PTB	UBN
BestKeeper	GAPDH	ACT	ZMPP	PP2A	EF	Fbox	UBC	PTB	UBN
Comprehensive	ACT	ZMPP	PP2A	GAPDH	UBC	Fbox	EF	PTB	UBN
Ranking order under salinity-alkalinity stress									
geNorm	UBC/UBN		Fbox	PP2A	ZMPP	ACT	GAPDH	PP2A	PTB
NormFinder	Fbox	PP2A	ZMPP	UBC	ACT	UBN	GAPDH	PTB	EF
BestKeeper	Fbox	ZMPP	PP2A	UBC	UBN	GAPDH	ACT	PTB	EF
Comprehensive	Fbox	UBC	PP2A	ZMPP	UBN	ACT	GAPDH	PTB	EF
Ranking order under drought stress									
geNorm	ACT/PTB		Fbox	ZMPP	UBN	PP2A	UBN	EF	GAPDH
NormFinder	PP2A	EF	PTB	Fbox	ZMPP	UBN	ACT	UBC	GAPDH
BestKeeper	EF	Fbox	PTB	ACT	ZMPP	UBN	UBC	PP2A	GAPDH
Comprehensive	PTB	Fbox	EF	ACT	ZMPP	PP2A	UBN	UBC	GAPDH

Stability of expression was then re-analyzed using the program BestKeeper, which is mainly through comparing the standard deviation (SD) and coefficient of variation (CV) of Ct value to select the most stable genes. The smaller SD and CV of reflect more stable reference gene (Table 3). For various cultivars, tissue samples, drought stress, inoculation treatment and salinity-alkalinity stress, the ranking generated by BestKeeper was slightly different from that determined by geNorm and NormFinder (Table 3). For waterlogging stress, *GAPDH* emerged as the most stably expressed.

### Comprehensive Stability Analysis of Reference Genes

To obtain a consensus result of the most stable reference genes as recommended by the three methods according to the RefFinder approach, the geometric mean of the three algorithms with the respective rankings for each candidate gene were calculated (Table 3). *PTB* was the most stable reference gene in different cultivars and drought stress. In the rust infection and waterlogging stress, *ACT* was most stable. Additionally, between different tissues, *UBN* was the best reference gene. And lastly *Fbox* was the most stably expressed under salinity-alkalinity stress.

### Evaluation of the expression of *CAT*, *GLU* and *CHI* by qRT-PCR

**Fig 4:**
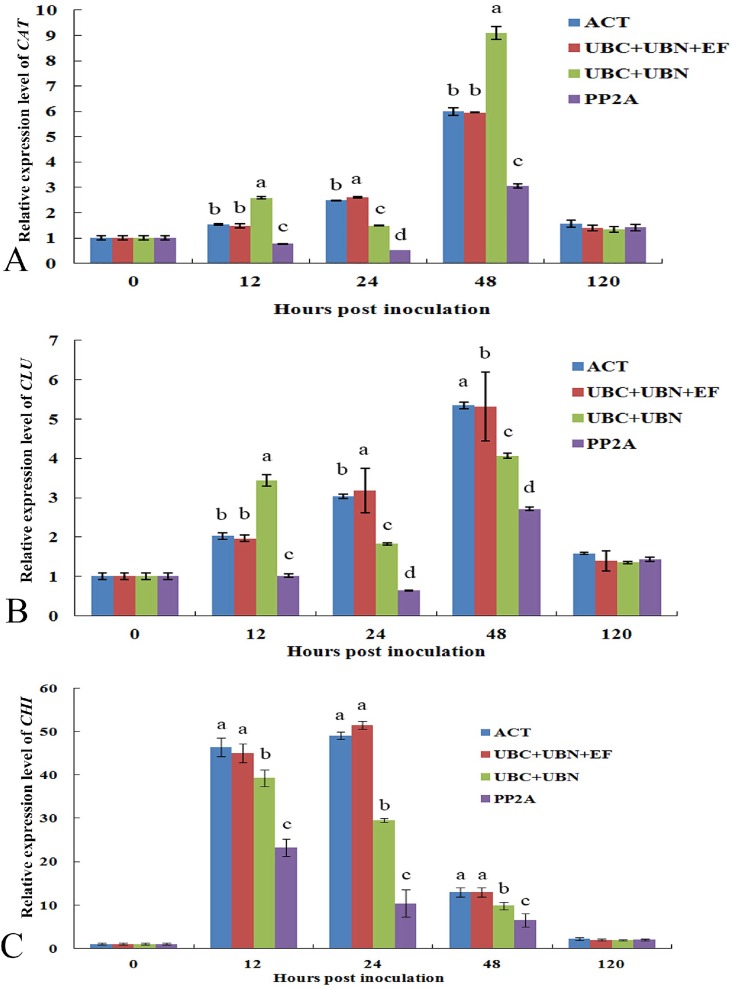
Relative quantification of *CAT*, *GLU* and *CHI* expression at different days after inoculation using validated reference genes for normalization. The validated reference gene(s), *ACT*, *UBC*+*UBN*+*EF*, *UBC*+*UBN* and *PP2A*, were used as normalization factors for analyzing catalase (*CAT*, A), chitinase (*GLU*, B) and β-1,3-glucanase (*CHI*, C) gene expression at different times of adzuki bean infected by *U*. *vignae*. *ACT* and *UBC*+*UBN*+*EF* were the most stable reference genes, while PP2A was the least stable. Results are presented as a mean fold change in relative expression compared to 0 h after inoculation.

To demonstrate the efficiency of the recognized reference genes in qRT-PCR, the expression of three genes (*CAT*, *GLU* and *CHI*) was analyzed during different times of *U*. *vignae* infection in BQH cultivar. The relative expression level of these genes were normalized by one or three most stable reference gene(s), and the least stable gene or two-gene combination. For *U*. *vignae* infection, the relative expression level of *CAT*, *GLU* and *CHI* showed no significant differences using either *ACT* alone or the combination *UNC*+*UBN* and *UBC*+*UBN*+*EF* as reference genes (Fig 4). However, the least stable reference gene(s) *PP2A* led to an underestimation of *CAT*, *GLU* and *CHI* transcript level. These results further confirmed the importance of the stability of reference gene, which could effectively reduce the occurrence of low precision or unreliable results.

## Discussion

Molecular detection and quantification of transcript abundance using qRT-PCR under different conditions are important tasks for gene function verification. However, the deviation of qRT-PCR are commonly introduced during RNA extraction, cDNA synthesis, and PCR process. Therefore, a suitable reference gene(s) for the specific set of chosen experimental conditions needs to be screened. An ideal reference gene should satisfy three norms: stable expression in test samples, similar amplification efficiency to target genes, and moderate level of expression.

Although *ACT*, *GAPDH*, *EF*, or 18s rRNA have been reported as reference genes for gene expression analysis [30–32], adzuki bean is not involved in these studies. Therefore, nine genes were selected as candidate reference genes in current study and three excel-based approaches BestKeeper, geNorm and NormFinder were used to evaluate the expression stability. Although there were distinct features among statistical algorithms and analysis strategies, these three approaches provided similar results. However, in some cases the gene expression stability was inconsistent among different softwares, such as *ACT*, *EF* and *Fbox* in waterlogging and salinity-alkalinity treatment, respectively. In this case, we selected a combination of two genes with most stable performances as reference. For example, both *UBC* and *UBN* were chosen as reference genes in salinity-alkalinity stress (Fig 3E), and both *ACT* and *ZMPP* as reference genes in waterlogging stress (Fig 3F). In addition, BestKeeper [33] and a comprehensive stability analysis [29] were used to obtain a consensus result of the most stable reference genes. Therefore, in the current study, a comprehensive analysis was used to confirm the most stable reference gene(s). However, no single reference gene had a consistent expression level in different experimental conditions. Compared with a single reference gene, selecting two or three stable reference genes can obtain more accurate and reliable data.

In the present study, we normalized the expression of *CAT*, *GLU* and *CHI* with a total of four normalization factors using individual (*ACT*) and combination of three (*UBC*+*UBN*+*EF*) control genes acquired a similar expression patterns. In contrast, when we normalized the expression of *CAT*, *GLU* and *CHI* using the most unstable reference gene, we acquired inconsistent result. These results indicated that reference genes screened in current study are solid and useful.

Actins play vital roles in cell motility and cytoskeleton maintenance, which has been confirmed to be suitable for gene expression normalization in legume crops [11] but not suitable for rice, potato or sugarcane [34, 35]. In current study, *VaACT* was the most stable genes among different conditions including different cultivars, biotic stress and drought stress [11]. Compared with *ACT*, *GAPDH* has been considered unstable among the most commonly used reference genes [29, 36]. Similarly, *GAPDH* was confirmed to be the most unstable gene in present study among all of the sample pools by both geNorm, NormFinder and BestKeeper analysis.

In the present study, nine housekeeping genes selected based on the whole genome sequences of *V*. *angularis* and among which the most stable reference genes were validated under different experimental conditions. However, with the development of high-throughput sequencing technology, we can also select stable reference genes from transcriptome profiling. For example, Wei *et al*. [12] exploited the transcriptome data to search the candidate reference genes of *P*. *massoniana* and picea for gene expression analysis. Nevertheless, without the transcriptome data in adzuki bean, the reference genes selected in current study will be helpful for accurate normalization of qRT-PCR data and facilitate the future work on gene expression studies in *V*. *angularis*.

## Conclusion

We have shown a set of stable housekeeping genes that are suitable to be used as reference genes in *Vigna angularis*. Different housekeeping genes responded with varying stabilities under various stresses. We found suitable reference genes among cultivars (*ACT* and *PTB*), various tissues (*EF* and *UBN*), and challenges including rust infection and drought stress (*ACT* and *ZMPP*), salinity-alkalinity stress (*UBC* and *Fbox*), and waterlogging stress (*Fbox* and *PTB*). We have proved that this is an accurate and reliable method for the normalization of qRT-PCR data in adzuki bean.

## Supporting Information

S1 Table Quality inspection of the total RNA using the Nanodrop 2000. (DOC)

S2 Table Ranking of the candidate reference genes according to their stability value using GeNorm. (DOC)

S3 Table Ranking of the candidate reference genes according to their stability value using NormFinde. (DOC)

S4 Table Ranking of the candidate reference genes according to their stability value using BestKeeper. Notes: Descriptive statistics of 9 candidate genes based on the coefficient of variance (CV) and standard deviation (SD) of their Ct values were determined using the whole data set. Reference genes were identified as the most stable genes, those with the lowest coefficient of variance and standard deviation.
(DOC)
