# Supplementary material for: Selection and Validation of Reference Genes for Gene Expression Analysis in Vigna angularis Using Quantitative Real-Time RT-PCR
Source: PLoS One. 2016 Dec 16;11(12):e0168479. doi: 10.1371/journal.pone.0168479 (PMC5161372; doi:10.1371/journal.pone.0168479)
Supplement: S1 Table — (DOC) [file pone.0168479.s001.doc]

**S1 Table. Quality inspection of the total RNA using the Nanodrop 2000**

| RNA samples | Concentration (ng/µL） | A260/A280 | A260/A230 |
| --- | --- | --- | --- |
| BQH | 593.55 | 1.90 | 2.03 |
| NAH | 975.00 | 1.94 | 2.12 |
| ZXC136 | 964.25 | 1.89 | 2.08 |
| ZXC143 | 531.65 | 1.91 | 2.14 |
| root | 932.50 | 1.83 | 2.12 |
| stem | 558.69 | 2.04 | 2.02 |
| fully expanded euphylla | 1073.45 | 1.85 | 2.28 |
| Drought stress 0dpi | 605.25 | 1.91 | 2.11 |
| Drought stress 6dpi | 540.85 | 1.84 | 2.01 |
| Drought stress 12dpi | 540.85 | 2.01 | 2.25 |
| salinity-alkalinity stress 0dpi | 300.65 | 2.18 | 2.05 |
| salinity-alkalinity stress 6dpi | 500.00 | 1.87 | 2.12 |
| salinity-alkalinity stress 12dpi | 37.16 | 1.83 | 2.05 |
| waterlogging stress 0dpi | 615.25 | 1.85 | 2.06 |
| waterlogging stress 6dpi | 521.85 | 1.89 | 2.04 |
| waterlogging stress 12dpi | 508.45 | 2.11 | 2.07 |
| biotic stress 0dpi | 756.75 | 2.03 | 2.01 |
| biotic stress 6dpi | 589.85 | 2.02 | 2.15 |
| biotic stress 12dpi | 536.90 | 1.84 | 2.18 |
