# Supplementary material for: Selection and Validation of Reference Genes for Gene Expression Analysis in Vigna angularis Using Quantitative Real-Time RT-PCR
Source: PLoS One. 2016 Dec 16;11(12):e0168479. doi: 10.1371/journal.pone.0168479 (PMC5161372; doi:10.1371/journal.pone.0168479)
Supplement: S2 Table — (DOC) [file pone.0168479.s002.doc]

**S2 Table.** Ranking of the candidate reference genes according to their stability value using GeNorm

| rank | Different conditions | | Different tissue | | Inoculate stress | | Waterlogging stress | | Salinity-alkalinity stress | | Drought stress | |
| --- | --- | --- | --- | --- | --- | --- | --- | --- | --- | --- | --- | --- |
|  | Gene | M value | Gene | M value | Gene | M value | Gene | M value | Gene | M value | Gene | M value |
| 1 | PTB | 0.548 | Fbox | 1.057 | ACT | 0.850 | PP2A | 0.836 | Fbox | 0.903 | Fbox | 1.057 |
| 2 | ACT | 0.586 | UBN | 1.134 | ZMPP | 0.973 | UBC | 0.907 | EF | 0.903 | UBN | 1.134 |
| 3 | PP2A | 0.602 | ZMPP | 1.139 | UBC | 0.974 | Fbox | 0.971 | UBC | 1.115 | ZMPP | 1.139 |
| 4 | UBC | 0.614 | ACT | 1.235 | Fbox | 1.015 | ACT | 1.111 | ZMPP | 1.148 | ACT | 1.236 |
| 5 | Fbox | 0.623 | PTB | 1.266 | UBN | 1.105 | ZMPP | 1.216 | UBN | 1.215 | PTB | 1.266 |
| 6 | ZMPP | 0.794 | UBC | 1.310 | PTB | 1.129 | EF | 1.235 | ACT | 1.427 | UBC | 1.531 |
| 7 | EF | 0.836 | EF | 1.734 | GAPDH | 1.146 | GAPDH | 1.426 | GAPDH | 1.821 | EF | 1.734 |
| 8 | UBN | 0.889 | PP2A | 1.794 | EF | 1.195 | PTB | 1.562 | PP2A | 1.931 | PP2A | 1.794 |
| 9 | GAPDH | 1.117 | ACT | 1.932 | PP2A | 1.615 | UBN | 1.863 | PTB | 2.126 | GAPDH | 1.822 |
