# Supplementary material for: Selection and Validation of Reference Genes for Gene Expression Analysis in Vigna angularis Using Quantitative Real-Time RT-PCR
Source: PLoS One. 2016 Dec 16;11(12):e0168479. doi: 10.1371/journal.pone.0168479 (PMC5161372; doi:10.1371/journal.pone.0168479)
Supplement: S3 Table — (DOC) [file pone.0168479.s003.doc]

**S3 Table.** Ranking of the candidate reference genes according to their stability value using NormFinde

| rank | Different conditions | | Different tissue | | Inoculate stress | | Waterlogging stress | | Salinity-alkalinity stress | | Drought stress | |
| --- | --- | --- | --- | --- | --- | --- | --- | --- | --- | --- | --- | --- |
|  | Gene | Stability | Gene | Stability | Gene | Stability | Gene | Stability | Gene | Stability | Gene | Stability |
| 1 | Fbox | 0.004 | EF | 0.021 | ACT | 0.228 | ACT | 0.440 | Fbox | 0.114 | PP2A | 0.012 |
| 2 | PTB | 0.033 | UBN | 0.043 | ZMPP | 0.309 | ZMPP | 0.507 | PP2A | 0.114 | EF | 0.020 |
| 3 | ACT | 0.035 | Fbox | 0.079 | Fbox | 0.338 | PP2A | 0.515 | ZMPP | 0.268 | PTB | 0.029 |
| 4 | PP2A | 0.035 | PP2A | 0.080 | PTB | 0.690 | UBC | 0.770 | UBC | 0.427 | Fbox | 0.111 |
| 5 | UBC | 0.064 | ZMPP | 0.130 | UBC | 0.802 | GAPDH | 0.859 | ACT | 0.469 | ZMPP | 0.159 |
| 6 | EF | 0.114 | UBC | 0.159 | GAPDH | 0.863 | Fbox | 1.084 | UBN | 0.608 | UBN | 0.202 |
| 7 | UBN | 0.118 | ACT | 0.175 | UBN | 1.048 | EF | 1.282 | GAPDH | 1.338 | ACT | 0.262 |
| 8 | ZMPP | 0.184 | GAPDH | 0.236 | EF | 1.097 | PTB | 1.765 | PTB | 2.252 | UBC | 0.297 |
| 9 | GAPDH | 0.344 | PTB | 0.256 | PP2A | 2.520 | UBN | 3.869 | EF | 3.514 | GAPDH | 0.370 |
