# Supplementary material for: Selection and Validation of Reference Genes for Gene Expression Analysis in Vigna angularis Using Quantitative Real-Time RT-PCR
Source: PLoS One. 2016 Dec 16;11(12):e0168479. doi: 10.1371/journal.pone.0168479 (PMC5161372; doi:10.1371/journal.pone.0168479)
Supplement: S4 Table — Notes: Descriptive statistics of 9 candidate genes based on the coefficient of variance (CV) and standard deviation (SD) of their Ct values were determined using the whole data set. Reference genes were identified as the most stable genes, those with the lowest coefficient of variance and standard deviation. (DOC) [file pone.0168479.s004.doc]

**S4 Table. Ranking of the candidate reference genes according to their stability value using BestKeeper**

| Treatments |  | ACT | Fbox | ZMPP | GAPDH | EF | PP2A | UBC | UBN | PTB |
| --- | --- | --- | --- | --- | --- | --- | --- | --- | --- | --- |
| Different cultivars  n=4 | SD [±Cq] | 0.800 | 1.230 | 0.990 | 1.650 | 1.140 | 1.140 | 0.880 | 0.690 | 1.030 |
|  | CV[%Cq] | 2.920 | 4.250 | 3.220 | 6.760 | 3.950 | 3.950 | 2.820 | 2.600 | 3.280 |
|  | Coeff. of corr.[r] | 0.999 | 1.000 | 0.913 | 0.910 | 0.984 | 0.984 | 0.969 | 0.900 | 0.993 |
| Different tissue  n=3 | SD [±Cq] | 2.000 | 0.940 | 1.280 | 1.510 | 1.000 | 1.380 | 1.510 | 1.280 | 0.880 |
|  | CV[%Cq] | 7.940 | 3.280 | 4.690 | 6.050 | 4.770 | 5.080 | 5.260 | 5.070 | 2.970 |
|  | Coeff. of corr.[r] | 0.829 | 0.997 | 0.874 | 0.806 | 0.999 | 1.000 | 0.887 | 0.998 | 0.818 |
| Inoculate treatmen  n=3 | SD [±Cq] | 1.150 | 0.800 | 0.530 | 1.000 | 1.680 | 0.710 | 1.420 | 1.580 | 0.340 |
|  | CV[%Cq] | 4.020 | 3.210 | 1.830 | 4.090 | 8.230 | 2.730 | 5.160 | 6.470 | 1.250 |
|  | Coeff. of corr.[r] | 0.994 | 0.895 | 0.999 | 0.836 | 0.999 | 0.229 | 0.988 | 0.988 | 0.987 |
| Waterloggingstress  n=3 | SD [±Cq] | 1.280 | 2.240 | 1.180 | 2.560 | 2.660 | 1.990 | 1.720 | 3.110 | 1.980 |
|  | CV[%Cq] | 4.640 | 8.400 | 4.080 | 9.870 | 12.160 | 7.590 | 6.330 | 11.73 | 6.240 |
|  | Coeff. of corr.[r] | 0.901 | 0.925 | 0.887 | 1.000 | 0.963 | 0.963 | 0.892 | 0.207 | 0.445 |
| Salinity-alkalinity stress n=3 | SD[±Cq] | 1.000 | 1.400 | 0.730 | 2.190 | 2.830 | 1.220 | 1.550 | 1.670 | 2.110 |
|  | CV[%Cq] | 3.550 | 5.100 | 2.420 | 7.900 | 11.29 | 4.210 | 5.210 | 5.570 | 7.890 |
|  | Coeff. of corr.[r] | 0.727 | 1.000 | 0.996 | 0.864 | 0.003 | 0.988 | 0.955 | 0.947 | 0.178 |
| Drought stress  n=3 | SD [±Cq] | 2.580 | 2.220 | 2.480 | 1.060 | 0.990 | 3.290 | 1.410 | 1.990 | 2.790 |
|  | CV[%Cq] | 8.830 | 8.050 | 7.580 | 4.160 | 4.600 | 10.790 | 4.730 | 7.480 | 8.950 |
|  | Coeff. of corr.[r] | 0.989 | 0.998 | 0.997 | 0.931 | 0.993 | 0.999 | 0.929 | 0.988 | 0.999 |

Notes: Descriptive statistics of 9 candidate genes based on the coefficient of variance (CV) and standard deviation (SD) of their Cq values were determined using the whole data set. Reference genes were identified as the most stable genes, those with the lowest coefficient of variance and standard deviation.
